# Patient involvement in priority-setting for medical research: A mini review of initiatives in the rare disease field

**DOI:** 10.3389/fpubh.2022.915438

**Published:** 2022-07-19

**Authors:** Amelia Katirai, Atsushi Kogetsu, Kazuto Kato, Beverley Yamamoto

**Affiliations:** ^1^Department of Kyosei Studies (Critical Studies in Coexistence, Symbiosis and Conviviality), Graduate School of Human Sciences, Osaka University, Suita, Japan; ^2^Department of Biomedical Ethics and Public Policy, Graduate School of Medicine, Osaka University, Suita, Japan

**Keywords:** rare disease (RD), patient involvement, priority-setting, medical research, review

## Abstract

Patient involvement (PI) in determining medical research priorities is an important way to ensure that limited research funds are allocated to best serve patients. As a disease area for which research funds are limited, we see a particular utility for PI in priority-setting for medical research on rare diseases. In this review, we argue that PI initiatives are an important form of evidence for policymaking. We conducted a study to identify the extent to which PI initiatives are being conducted in the rare disease field, the features of such initiatives, the trends in the priorities elicited, and the extent to which translation into policy is reported in the academic literature. Here, we report the results of this exploratory review of the English-language literature gathered through online databases and search engines, with the aim of identifying journal articles published prior to December 2020, describing PI initiatives focused on determining priorities for medical research funding in the rare disease field. We identified seven recently-published articles and found that the majority made use of structured methodologies to ensure the robustness of the evidence produced, but found little reported practical implementation or concrete plans for implementation of the results of the initiatives. We conclude that priority-setting initiatives are meaningful mechanisms for involving patients in determining research directions. However, we highlight the importance of translation into policy as a necessary next step to fully utilize the results and move beyond well-intentioned exercises. Finally, we draw attention to the benefits of involving patients throughout this process.

## Introduction

There is a growing consensus about the need to involve patients throughout the medical research process, beginning with priority-setting for medical research policy. Patient involvement (PI) in priority-setting is a way to ensure that limited research funds are allocated in ways that best serve patients, and is based on the principle that patients' lived experiences provide unique expertise which is critical to robust policy directions (1–4). Here, we use Schilling et al. (5) definition of patients as those who “bring specific health related experiences into research as members of the public,” and may include caregivers, family, friends, or other significant people in the lives of patients.

Given the limited availability of funds for research on rare diseases, ensuring that available funds are appropriately allocated is critical (6, 7). Priority-setting initiatives involving patients may have particular utility in the field of rare diseases, yet have been often overlooked in reviews of the field (8). To remedy this, we report the results of an exploratory review of the literature on priority-setting initiatives focused on rare diseases that involve patients. We argue that ensuring that the outcomes of these initiatives are translated into policy is critical, and that viewing the results of these outcomes as evidence can support this.

## Patient involvement in determining priorities for medical research on rare diseases

Although orphan drug laws have strengthened the incentives for investment in the area of rare diseases, the amount of funding available for medical research remains “disproportionately limited” (9), given the estimated 350 million individuals globally affected by over 7,000 recognized rare diseases (6, 7, 10). There is also disparity in funding between different rare diseases, with ultra-rare diseases and those with early onset, as particularly underserved (7).

PI through priority-setting exercises is one way to determine how to allocate available resources, given limited funds. Often conceptualized in tandem with public involvement as patient and public involvement, it “is being adopted for diverse reasons from cost-containment and shifting responsibility to better tailoring of services to meet the needs of patients and communities” (11). It helps to remedy a lack of public input and the dominance of the pharmaceutical and medical devices industry in medical research, as identified by Buckley et al. (12). The results of priority-setting initiatives can have an impact on directions for funding and future research at the levels of policy, institutions, and research teams (13).

There is a growing recognition of the importance of ensuring that patients are given a greater say in medical care, in settings ranging from research and development to clinical care, as the incorporation of “patient voice … is becoming the expected norm and, in some instances, a requirement” (14) in many contexts. Notable examples from the United States include the establishment of Patient Listening Sessions by the Food and Drug Administration (FDA), through which patients and advocates are invited to speak to representatives of the FDA (15), and patient-focused drug development, where patients are asked to use their lived experience to share insights relevant to drug development, regulation, and clinical trials (16).

In the context of research, a key expectation is that PI will help to ensure that research pre-emptively addresses the needs of the end-users of healthcare (17, 18). It attempts to resolve mismatches between the priorities of research communities and industry, and the needs of patients and clinicians, preventing waste (10). Recent studies illustrate the importance of PI. For example, Crowe et al. (19) found that priorities elicited from patients and clinicians emphasized non-drug treatments, whereas research communities tended to focus on expanding drug trials. Similarly, a study by Fleurence et al. (20) found that the ongoing involvement of patients and other stakeholders in selecting proposals for funding led to the selection of a different set of proposals from what would have been chosen through peer review. Forsythe et al. (21) propose that other stakeholders should include “caregivers such as parents of children with rare diseases, advocacy organizations, and clinicians,” with the involvement of such diverse stakeholders leading to more effective and meaningful research.

Additionally, PI can provide conditions for patient empowerment. This is defined by Ayme et al. (1, 5) as, “an action-oriented notion with the focus on … transformation of relations between communities and institutions,” and particularly critical for rare disease patients who experience unmet needs and lack opportunities to have their voices heard. This makes PI distinct from public involvement, which is underpinned by the principle that citizens have a civic right to be democratically involved in decision-making (3). Patient empowerment is particularly valuable for rare diseases, which may be “chronic, difficult to manage, [and] so rare that coordinated efforts are imperative to make progress,” as Ayme et al. (1) further report. Involvement benefits patients by facilitating their active participation in a community and ensuring that their needs are attended to by policymakers, as will be discussed in the next section.

## PI as a source of evidence for policymaking

Alongside growing support for PI, the importance of evidence-based or evidence-informed (22) policymaking for medical research is increasingly recognized. This recognition has led to debate over what constitutes good evidence for policymaking—with a tendency to import hierarchical models used in evidence-based medicine that privilege randomized controlled trials (23). However, there is further debate about the appropriateness of this approach. For example, Parkhurst and Abeysinghe (23) criticize overreliance on randomized controlled trials as they may fail to appropriately account for other matters such as the social and human rights aspects of policy (24). Moreover, these authors argue that there is no universally applicable “best” kind of evidence as implied by these hierarchies. Rather, good evidence should be suited to the exigencies of the situation, draw on both qualitative and quantitative data, and include reports of experiences (25). Against this background, we focus here on PI initiatives as a method of evidence production for policymaking that can generate in-depth, qualitative, experiential data which offers a window into the social implications of policy, as argued by Rand et al. (26).

For the purposes of this paper, we use a conceptualization of good evidence as having: (1) credibility—being scientifically appropriate; (2) salience—being relevant to the needs of policymakers; and (3) legitimacy—being fair and respectful toward stakeholders in the data gathering process (27). To what extent, then, can PI initiatives be expected to produce robust evidence for policymaking? Credibility is increasingly furnished through structured mechanisms for patient involvement such as the James Lind Alliance's (JLA) Priority-Setting Processes (18). The JLA brings patients, caregivers, and clinicians together in Priority-Setting Partnerships, to identify and determine the priority of unanswered questions—evidence uncertainties—in a particular health area (18). PI initiatives have salience, as they are used to identify needs for funding, and ensure that policy outcomes directly reflect the concerns and priorities of the end-users of treatments (19). And finally, PI has both input and output legitimacy by ensuring broader participation in policymaking and that issues of importance to patients are reflected (3).

Patient involvement and public involvement more broadly are often conceptualized as existing on a continuum, ranging from consultation to partnership and shared leadership, though initiatives often remain situated on the shallow end of this spectrum, where patients “are involved but have limited power or decision-making authority” (28, 29). Thus, PI is at risk of being “tokenistic, narrow and exclusive” (28), conducted to increase the *perceived* legitimacy of evidence, and perpetuating a “fund and forget model" (30, 31). PI initiatives reflect this model in particular when insufficient support and other structural issues prevent the outcomes of initiatives from being utilized as the basis for future decision-making. However, this model can be avoided through a focus on ensuring that the initiative can be sustained over time. Jinks et al. (32) suggest that this can be achieved through an approach based on leadership which promotes the value of PI initiatives, the provision of sufficient resources, and the development of appropriate infrastructure, including staff charged with supporting the relationships which form the basis for PI.

Moreover, as Fredriksson and Tritter (3) argue, the issue of representativeness is a common obstacle to the utilization of the evidence produced through PI initiatives. Initiatives are resource-intensive for those conducting them and for those participating, and generally involve a small subset of a larger population of interest (3, 4, 28). As open-door arenas, the unique positioning of patients involved may create particular and pressing concerns that are not representative of broader populations (3, 29). However, these limitations are offset by understanding PI as a technocratic, rather than democratic, exercise, as proposed by Martin (33). Through this understanding, PI does not need to involve representative patients, but rather to draw out the expertise of those who are experts in their own conditions. Therefore, a lack of representativeness does not diminish the value of the evidence produced. Moreover, small-scale initiatives can set off a ripple effect that draws in further financial resources to access hard-to-reach participants.

A tendency in PI is for initiatives to be “limited to preliminary activities that are not sustained across the research activity lifecycle” (2). A recent study by Staley et al. (13) found that few JLA initiatives were able to successfully translate the outcomes of initiatives into policy directions. This prevents resource maximization, and may undermine patient empowerment when meaningful changes to policy do not result from PI. For this reason, we argue that the full involvement of rare disease patients in the policy-making process requires their involvement not only in consultative exercises, but also into dissemination and advocacy for implementation in policy. In the sections below, as we report the findings of a review of the literature identifying priority-setting initiatives in the rare disease field, we examine the extent to which patients are reported to be involved throughout this extended process.

## Methodology

In this review, we take as our starting point the observation from Mickute et al. (8) that only four of the over one hundred recent JLA Priority-Setting Processes (PSPs)—one of the key structured methods for PI—were focused on rare diseases. We conducted an exploratory review of the literature through the online databases Scopus, ProQuest, and Semantic Scholar, supplemented by a Google Scholar search (34), with the aim of identifying journal articles describing PI initiatives identifying priorities for medical research funding in the area of rare diseases. Articles describing PI but unrelated to priority-setting were excluded. Literature was gathered through variations of the keywords: rare diseases/cancers, patient engagement/involvement, and priority-setting. Data collection was limited to English language literature. Any identified articles published before December 2020 were included in the review.

## Results

The results of our review are charted in (Table 1). In addition to the four publications identified by Mickute et al. (8), we identified three recent articles documenting PI initiatives in priority-setting, for a total of seven published initiatives. These were in the areas of pulmonary fibrosis (40), musculoskeletal diseases (8), liver glycogen storage disease (39), cystic fibrosis (37), idiopathic intracranial hypertension (36), and dystrophic epidermolysis bullosa (35). One study (38) was concerned with priority-setting across multiple rare diseases. Two studies were concerned with ultra-rare conditions with a prevalence under 1 in 50,000 (35, 40).

**Table 1:** Included articles.

**Citation**	**Davila-Seijo et al. (35)**	**Mollan et al. (36)**	**Rowbotham et al. (37)**	**Somanadhan et al. (38)**	**Peeks et al. (39)**	**Mickute et al. (8)**	**Tikellis et al. (40)**
Area	Dystrophic epidermolysis bullosa	Idiopathic intracranial hypertension	Cystic fibrosis	Rare diseases	Liver glycogen storage disease	Musculoskeletal diseases	Pulmonary fibrosis
Ultra-rare	Yes	No	No	(Not applicable)	No	No	Yes
Methodology	JLA	JLA and NGT	JLA (Adapted)	Participatory multiple method and priority-setting partnerships	JLA	JLA	JLA and NGT
Condition	Single	Single	Single	Multiple	Single	Multiple	Single
Setting	Spain	UK	Online (Based in UK)	Ireland	The Netherlands	UK	Australia

The total number of participants in the above processes ranged from 88 to 763, and included patients, caregivers, and healthcare professionals or other experts. The proportion of patients and caregivers among participants in the priority-setting ranged from 23 percent to 85 percent, where reported. Participants were generally recruited through the contacts of steering committees, which had ties to patient organizations or used online channels, including advertising through social media and email.

The majority of the studies made use of the James Lind Alliance (JLA) methodology, highlighting its primacy as an effective mechanism for involving patients. The Nominal Group Technique (NGT), a “consensus method” which involves stakeholders in discussion, and aims to have all voices heard equally was also used (41). Due to the structured nature of the methodologies the resulting roles of patients in the studies were generally similar: they were present in the steering committees and were involved in the first four steps of the JLA process.

Overall, there was little reported practical implementation or concrete plans for the implementation of the results of the processes, though almost all of the authors (8, 35–40) expressed hopes that botd researchers or funding bodies would take up the results of the initiatives and use them to inform future funding decisions. Rowbotham et al. (37) reported immediate dissemination of the results through social media and a press release, while also informing key stakeholders and consulting witd the NIHR. However, no studies reported concrete actions to involve patients in dissemination, and though patients may have been represented in steering committees involved in the dissemination of results, this was not clearly delineated.

We also tabulated and examined the 77 priorities reported across the articles (Table 2). Collectively, the largest number of priorities reported in the studies were concerned witd treatment methods (*n* = 28), including which treatments would lead to better outcomes, and investigation into alternative and/or novel treatments. Identifying the benefits and side effects of particular treatments were also priorities in this area. Symptom management (*n* = 11) was also a key area of concern. Ten of the priorities reported in the studies were concerned witd the psycho-social impact of the disease, including the need for support at diagnosis and at significant life transitions, the impact of the disease not only on patients but also on family members and significant others, and the economic impact of having the disease. Other priorities related to prevention, (*n* = 8), the causes of the diseases (*n* = 7), prevention (*n* = 7), diagnosis (*n* = 5), prognosis and mechanisms through which to prevent the progression of the disease and/or associated healtd concerns (*n* = 4). Priorities about patient voice—how to elicit patient voice in care and regarding mechanisms for co-design—appeared as well (*n* = 2), while priorities regarding how to better educate and inform healthcare professionals about rare diseases (*n* = 1), and the establishment of mechanisms for data sharing were also present (*n* = 1).

**Table 2:** Priorities identified in the studies.

**Code**	**Totals**	**Proportion**
Treatment	28	36%
Symptom management	11	14%
Psycho-social impact	10	13%
Prevention	8	10%
Causes	7	9%
Diagnosis	5	6%
Prognosis	4	5%
Patient voice	2	3%
Education	1	1%
Data sharing	1	1%
Total	77	100%

## Discussion

Despite the vast number of rare diseases, the large global population impacted, and the sparsity of research funding, there are few PI initiatives on rare diseases. However, we see the recent gradual increase in the number of published initiatives as a promising sign that investments are being made for the voices of patients to be better heard in policymaking. It is encouraging that these processes can involve hundreds of participants. In addition, the use of structured methodologies such as the JLA or NGT methods ensure the robustness of the evidence produced (26). Building on these strengths, we propose improvements to the process and reporting of PI initiatives identified through this study.

The lack of reporting on practical plans for the implementation of the outcomes of PI initiatives identified is a tendency which extends beyond the rare disease field, as described above (2, 13). For this reason, we propose that plans for PI initiatives should include measurable steps to ensure that the results are translated into policy. Moreover, this should be reflected in the reporting of initiatives, which should include a description of practical progress toward the implementation of results. Given the findings of a recent systematic review that involving patients in dissemination is beneficial, we also highlight the importance of visibly involving patients in dissemination (42).

As discussed above, the outcomes of PI initiatives meet the criteria for good evidence for policymaking: credibility, salience, and legitimacy, proposed by Parkhurst (27). In light of this, we echo Rand et al. (26) in arguing that explicitly using the concept of evidence in framing the results of PI initiatives would help to position them in the broader context of evidence-based policymaking, raising the profile of the results. This could be supported by greater transparency regarding the demographic characteristics of patients involved, which would strengthen the quality of the evidence produced.

Furthermore, our analysis showed that the psycho-social impact of rare diseases was a recurrent and important topic in the results of these initiatives. This is experienced firsthand by patients and caregivers, but only secondhand by clinicians, who often focus on the disease itself rather than the issues patients may experience in daily life. It is even further at a distance from researchers or industry actors and may be perceived to be beyond the usual framework of biomedical research. These are issues that patients are particularly attuned to, given their lived experience–and which can be brought to light by patients themselves through PI initiatives (26). Thus, our findings support the importance of involving patients in setting research directions.

Our findings also suggest that eliciting priorities from patients witd related rare diseases may be a useful strategy through which to ensure that greater numbers of patients are involved. This is particularly useful in settings where logistical barriers to participation or the small size of rare disease populations in a particular setting may be obstacles to conducting priority-setting focused around a single rare disease (38). However, the participation of patients from multiple rare diseases may shift attention away from disease-specific issues and toward identifying shared, system-level and quality-of-life-related issues. As Somanadhan et al. (38) indicate, “[r]are diseases are individually unique, but collectively they share substantial unmet healtd and social care needs.” However, a risk through this approach is that some of the nuance in relation to particular conditions be lost through the process, and result in compromises to achieve consensus.

The initiatives identified through this study were all centered in Western European settings where PI is more firmly established. However, in settings where PI is relatively new, drawing together patients witd a range of rare diseases in this way can ensure that enough patients can be involved. For example, Japan—where the authors of this paper are situated—is a country where PI is just beginning to gain traction witd key regulatory bodies promoting its use for medical research (43–45). Authors of this paper are seeking to elicit rare disease patient perspectives on medical research policymaking, and to involve patients in interactions witd policymakers through a project entitled “Constructing a commons utilizing ICT to generate evidence for medical policy”(46). This team has found that bringing together patients from multiple rare diseases ensures sufficient participation in order to elicit novel and meaningful insights into the experiences and priorities of rare disease patients.

In this way, our exploratory review of initiatives for patient involvement in policymaking has highlighted movements toward patient involvement in priority-setting in the rare disease field. Given the limited resources available for research on rare diseases, we propose that priority-setting initiatives involving patients are particularly meaningful. Although these initiatives are undoubtedly resource-intensive, they reflect an intrinsic commitment to ensuring that patient voices are heard (10). By highlighting initiatives that have successfully taken steps in this direction, we hope that we have given further impetus to these movements. However, in order to ensure that these initiatives are translated into policy outcomes that can benefit the patients involved, we maintain that appropriate next steps toward implementation should be embedded in any plans for action, and reported when results are disseminated.

### Limitations of the study and future directions

Due to the exploratory nature of this review, a flexible review methodology was selected. However, future research could expand on this study through a more systematic review of the field as it grows. Furthermore, this study examined the extent to which PI initiatives were reported in the academic literature, but did not include within its scope reports from other sources. Further research could include an exploration of the gray literature. Perhaps most importantly, we noted in this study that few initiatives reported whether or not the results had been implemented. Future research could focus on the extent to which priority-setting initiatives in the rare disease field have influenced policy decisions and had an impact on research funding priorities. It could also take within its scope the importance of PI initiatives at multiple levels of the policy-making process, including considerations of the potential role for PI in the development and implementation of new legislation, and the infrastructure that could facilitate this.

## Author contributions

AKa, BY, AKo, and KK contributed to the design and implementation of the research and to the analysis of the results. AKa drafted the paper. All authors contributed to manuscript revision, and read and approved the submitted version.

## Funding

This work was supported by Japan Science and Technology Agency (JST) Research Institute of Science and Technology for Society (RISTEX) Grant Number JPMJRX18B3, Japan.

## Conflict of interest

The authors declare that the research was conducted in the absence of any commercial or financial relationships that could be construed as a potential conflict of interest.

## Publisher's note

All claims expressed in this article are solely those of the authors and do not necessarily represent those of their affiliated organizations, or those of the publisher, the editors and the reviewers. Any product that may be evaluated in this article, or claim that may be made by its manufacturer, is not guaranteed or endorsed by the publisher.
